# Exotic QTL improve grain quality in the tri-parental wheat population SW84

**DOI:** 10.1371/journal.pone.0179851

**Published:** 2017-07-07

**Authors:** Ioanna-Pavlina Nedelkou, Andreas Maurer, Anne Schubert, Jens Léon, Klaus Pillen

**Affiliations:** 1 Martin-Luther-University Halle-Wittenberg, Institute of Agricultural and Nutritional Sciences, Chair of Plant Breeding, Halle, Germany; 2 University of Bonn, Institute of Crop Science and Resource Conservation, Crop Genetics and Biotechnology Unit, Katzenburgweg 5, Bonn, Germany; Institute of Genetics and Developmental Biology Chinese Academy of Sciences, CHINA

## Abstract

**Developing the tri-parental exotic wheat population SW84:**

Genetic diversity of cultivated wheat was markedly reduced, first, during domestication and, second, since the onset of modern elite breeding. There is an increasing demand for utilizing genetic resources to increase genetic diversity and, simultaneously, to improve agronomic performance of cultivated wheat. To locate favorable effects of exotic wheat alleles, we developed the tri-parental wheat population SW84. The population was derived from crossing the hexaploid spring wheat cultivars Triso and Devon with one synthetic exotic donor accession, Syn084L, followed by two rounds of backcrossing and three rounds of selfing. SW84 consists of 359 BC_2_F_4_ lines, split into two families, D84 (Devon*Syn084L) and T84 (Triso*Syn084L).

**Studying the genetic control of grain quality in SW84:**

As a case study, grain quality of SW84 was studied in replicated field trials. Transgressive segregation was observed for all studied grain quality traits by evaluating SW84 for two years at two locations under low and high nitrogen supply. Subsequently, a genome-wide association study (GWAS) was carried out based on genomic data derived from a 90k Infinium iSELECT single nucleotide polymorphism (SNP) array. In total, GWAS yielded 37 marker-trait associations, summarized to 16 quantitative trait loci (QTL). These SNPs indicate genetic regulators of grain protein content, grain hardness, sedimentation value and sedimentation ratio. The majority of exotic QTL alleles (75%) exerted favorable effects, increasing grain protein content and sedimentation value in ten and two cases, respectively. For instance, two exotic QTL alleles were associated with a substantial increase of grain protein content and sedimentation value by 1.09% and 7.31 ml, respectively. This finding confirms the potential of exotic germplasm to improve grain quality in cultivated wheat. So far, the molecular nature of most of the detected QTL is unknown. However, two QTL correspond to known genes controlling grain quality: The major QTL on chromosome 6B, increasing grain protein content by 0.70%, on average, co-localizes with the *NAM-B1* gene, known to control grain protein content as well as iron and zinc content. Likewise, the major QTL on chromosome 5D, reducing grain hardness by 8.98%, on average, co-localizes with the gene for *puroindoline b (Pinb-D1)* at the *Ha* locus. In total, 13 QTL were detected across families, whereas one and three QTL were exclusively detected in families D84 and T84, respectively. Likewise, ten QTL were detected across nitrogen treatments, whereas one and five QTL were exclusively detected under low and high N treatments, respectively. Our data indicate that most effects in SW84 act across families and N levels. Merging of data from two families or two N treatments may, thus, be considered in association studies to increase sample size and, as a result, QTL detection power.

**Utilizing favorable exotic QTL alleles in wheat breeding:**

Our study serves as a model how favorable exotic QTL alleles can be located in exotic germplasm of wheat. In future, the localized favorable exotic QTL alleles will be utilized in wheat breeding programs to simultaneously improve grain quality and selectively expand genetic diversity of the elite wheat gene pool.

## Introduction

Wheat (*Triticum aestivum*) is one of the most important crops in the world and the most widely grown cereal, covering 220 million hectares of land and ranking third with a production of 729 million tons in 2014 [1]. It is the source of up to 55% of carbohydrates consumed worldwide [2] and supplies more than 60% of calories and proteins for human nutrition [3]. Cultivated hexaploid wheat (2n = 6x = 42, BBAADD) is derived from spontaneous hybridization of cultivated tetraploid emmer, *Triticum turgidum* ssp. *dicoccum* (2n = 4x = 28, BBAA), with diploid *Aegilops tauschii* (2n = 2x = 14, DD) [4].

It is widely accepted that genetic diversity in wild relatives of wheat is considerably higher than in cultivated wheat. Exotic wheat accessions, thus, have a potential to assist in improving grain quality [5] by contributing favorable alleles that control grain protein content [6], [7] and other grain quality traits [8]. Also, Nevo [9] valued cultivars possessing wild emmer genes as promising, due to its reservoir of resistance genes against pathogens (e.g. powdery mildew) and genes conferring favorable effects on protein content and baking quality [10]. In addition, *Aegilops tauschii* confers grain softness to bread wheat via its D genome contributing to bread-making quality [11].

Hexaploid soft wheat exhibits a relatively low grain protein content, ranging from 8 to 15%, however, in less developed countries grain protein content and the nutritional quality of cereals may be of critical importance to prevent human malnutrition [12, 13]. Grain protein content affecting the nutritional value and the baking properties of common wheat is also important for market grading and classification [14] and determines the pasta-making characteristics of durum wheat (*Triticum turgidum* var. *durum*) as well as bread-making quality and dough quality of soft wheat [15]. Grain protein content is a typical quantitative character controlled by multiple genetic factors and strongly influenced by environmental conditions and management practices such as nitrogen fertilization, water availability, temperature and light intensity [16]. It has been reported that grain protein content in soft wheat can be increased up to 16–19% after conventional gene transfer from wild emmer [5]. Thus, wild wheat, in particular wild emmer, is regarded as a valuable resource for increasing grain protein content in hexaploid wheat, itself reaching a grain protein content of up to 28% [10].

Several studies have shown that quantitative trait loci (QTL) explaining variation in grain protein content in cultivated and wild wheat are located on different wheat chromosomes [17], [18], [19], [20], [21], [22], [23], [24], [16], [25], [26], [27]. Usually, grain protein content is negatively correlated with grain yield [28], [24], [29] and, thus, breeding for improvement of this trait proved to be difficult. Increases in grain protein content have been achieved via N supply [16], [29]. Nevertheless, the increasing cost of fertilization together with the environmental concern associated with fertilization [30] may limit N fertilization in the near future. A wild emmer wheat, *T*. *turgidum* ssp. *dicoccoides*, accession, from Israel exhibiting a high grain protein content was the source to map the grain protein content QTL *Gpc-B1* on the short arm of chromosome 6B [31]. Positional cloning of this gene [6] showed that *GPC-B1* belongs to the *Arabidopsis* NAC transcription factor family. The gene accelerates senescence and, simultaneously, increases the content of grain protein, zinc and iron [32] by influencing translocation of the available N [33] as well as other nutrients from vegetative tissue to grains [22].

Grain hardness and sedimentation volume are further important grain quality traits in bread wheat. Grain hardness is negatively correlated with milling yield, has an impact on bread quality [34] and strongly influences end-use quality such as size and shape of flour particles. Genes for grain hardness are present at the grain hardness (*Ha*) locus on wheat chromosome arm 5DS, controlling grain texture. Closely linked to the *Ha* locus are genes for grain softness and friabilins, namely puroindoline a (*Pina*), puroindoline b (*Pinb*) and the grain softness related protein (*Gsp-1*) locus. Wild species alleles (*Pina-D1a*, *Pinb-D1a*) encode soft endosperm, while mutations in *Pina* or *Pinb* resulted in a firm endosperm [35], [32], [36], [37]. In addition, [38] reported on anti-microbial properties of puroindolines.

Sedimentation volume is a measure of flour quality and a quality estimate of gluten strength. It is closely connected with high molecular weight glutenins and gliadins [19], [23], [39]. Studies report on strong positive correlation with baking quality, cooking quality, loaf volume, mixograph score and viscoelasticity of pasta [39]. Previous studies showed that the *Glu-1* and *Glu-3* loci on the long and short arms of homoeologous group 1 chromosomes, encoding high and low molecular weight glutenins, respectively, served as candidate genes to control sedimentation and baking quality [40], [41].

Efficient introgression strategies in crop breeding, such as advanced backcross quantitative trait locus (AB-QTL) analysis, allow to simultaneously detect QTL and develop varieties [42]. Besides tomato, this strategy has also been applied to barley [43], [44] and wheat [19], [45], [46], [47]. Also, the nested association mapping strategy, implemented by Yu et al. [48] and McMullen et al. [49] can be used in order to select new trait-improving QTL alleles from exotic donors [50, 51].

The tri-parental spring wheat population SW84, reported here, was developed to compare the effects of genetic diversity in two elite parent genetic backgrounds, following a combination of the advanced backcross and the nested association mapping approaches. SW84, comprising of 399 BC_2_F_4_ lines, was derived from crosses of the synthetic wild wheat donor accession Syn084L with the spring wheat cultivars Devon and Triso as recurrent parents. To study the genetic diversity present in SW84, single nucleotide polymorphism (SNP) markers were used. SNP markers are promising for wheat breeding due to their availability for high-throughput genotyping [52], [53], [54]. SNP genotyping methods, including high-density arrays like the 9k SNP wheat chip [55] and the 90k SNP wheat chip [54], are available for wheat and have been used in various mapping and QTL studies.

The objective of the present study was to characterize the tri-parental wheat population SW84 based on quality parameters measured under two N fertilization levels. QTL, effective across nitrogen treatments as well as restricted to individual nitrogen treatments and families and potentially valuable exotic QTL alleles are detected. In future, these QTL will assist to improve quality traits in wheat breeding and widen the genetic diversity of our modern elite wheat gene pool.

## Materials and methods

### Plant material

A BC_2_F_4_ population, SW84, derived from crossing two German spring wheat cultivars, Devon and Triso, as recurrent parents, with the synthetic hexaploid wild wheat accession Syn084L as the donor parent was developed for QTL analysis. Devon is characterized by a grain protein content of >12%. It was developed by Hans-Ulrich Hege GmbH & Co. KG, Waldenburg, Germany, and obtained from Monsanto Agrar Deutschland GmbH, Düsseldorf, Germany. Triso is characterized by a grain protein content of >14% and was developed and obtained by Deutsche Saatveredelung AG, Lippstadt, Germany. The synthetic wild wheat accession Syn084L was developed by hybridization of *Triticum turgidum* ssp. *dicoccoides* with *Aegilops tauschii* (Lange and [56, 57] and kindly provided by Dr. W. Lange, Centre of Genetic Resources (CPRO, Wageningen, The Netherlands). The recurrent parents Devon and Triso were used as female parent and the exotic donor Syn084L as male parent of the initial crosses. Per initial cross, a single maternal F1 plant was backcrossed with the respective cultivars. From each initial backcross, 27 Devon and 18 Triso maternal BC_1_F_1_ plants were backcrossed again with the respective cultivars. After three cycles of selfing the BC_1_F_1_ plants, 154 and 205 seeds of the Devon×Syn-84 backcross (i.e. family D84) and of the Triso×Syn-84 backcross (i.e. family T84), respectively, were randomly chosen to set up the BC_2_F_4_ population SW84. The seeds were bulk propagated for two further cycles in field plots resulting in a set of 359 BC_2_F_4:6_ lines of population SW84. These lines were subjected to phenotypic evaluation during the growing seasons 2004 and 2005.

### Genotyping population SW84

In 2013, DNA was extracted from 30 mg of fresh young leaf material of SW84 seedlings, grown in the greenhouse, following the procedure of Schmalenbach et al. [58]. For this, leaf material from 12 BC_2_F_4:6_ plants per SW84 line was pooled and homogenized using a TissueLyser bead mill (Qiagen N.V., Hilden, Germany) and isolated using the BioSprint DNA Plant Kit and the BioSprint 96 workstation from Qiagen. For Illumina SNP genotyping, a sample concentration of approximately 50 ng/μl and a sample volume of at least 20 μl was prepared. Genotyping of SW84 lines was conducted at Traitgenetics GmbH, Gatersleben, Germany, using the Illumina 90k wheat SNP chip. Chromosomal positions were assigned to SNPs according to the consensus map of Wang et al. [54].

### Phenotyping population SW84

Three hundred and fifty-nine lines of the tri-parental population SW84 were grown and evaluated under two nitrogen fertilization levels (N0 and N1) for quality traits at two locations, Dikopshof (University of Bonn) and Hovedissen (W. von Borries Eckendorf GmbH & Co. KG), and during two seasons, 2004 and 2005, resulting in three environments tested (D04, H04, H05). Among the environments plot sizes ranged from 3.75 (H04 and H05) to 6.00 m^2^ (D04) and seed density ranged from 400 (D04) to 430 seeds/m^2^ (H04 and H05). The soil N_min_ reached 44, 35 and 40 kg N/ha, the N0 treatment received an N fertilization of 35, 45 and 50 kg N/ha and the N1 treatment received an N fertilization of 150, 140 and 150 kg N/ha at environments D04, H04 and H05, respectively. Phosphate and potassium fertilization, growth regulator treatment and pest control treatments were the same for N0 and N1 blocks and followed local practice.

Grain protein content (GPC in %), grain hardness (GH in %) and sedimentation value (SED in ml) were determined using the Inframatic 8600 Flour Analyzer (Perten Instruments, Hamburg, Germany) and a near infrared reflectance (NIR) spectroscopy protocol [59]. The different performance of SW84 lines under both N levels were characterized as trait ratio from the following calculation: Trait ratio = Trait N0 / Trait N1, using LSMEANS across environments for calculation, as indicated below.

### Statistical analyses

Statistical analyses were conducted with SAS 9.4 Software (SAS Institute Inc., Cary, NC, USA). Chi-square tests (SAS procedure FREQ) were used to calculate segregation distortion, namely deviation from the expected ratio of 0.859: 0.031: 0.109 (homozygous elite to heterozygous to homozygous exotic genotypes) in BC_2_F_4_. SNPs deviating significantly from the expected single gene segregation ratio were discarded from QTL analysis to avoid wrong genotype calling. Least squares means (LSMEANS) across environments, within and across N levels, were calculated with SAS procedure MIXED in order to calculate adjusted means, range, standard deviation and coefficient of variation for each trait. The LSMEANS per line were subsequently used to calculate genetic correlations (Pearson correlation coefficient, r) between traits with SAS procedure CORR. Trait heritabilities were estimated across environments and N levels as:

$$h^{2}=\frac{V_{G}}{V_{G}+\frac{V_{GxE}}{e}+\frac{V_{GxN}}{n}+\frac{V_{R}}{e\times n}}\;\mathrm{for}\;\mathrm{GPC},\;\mathrm{GH}\;\mathrm{and}\;\mathrm{SED},$$

and within N levels as:

$$h^{2}=\frac{V_{G}}{V_{G}+\frac{V_{R}}{e}}\;\mathrm{for}\;\mathrm{GPC},\;\mathrm{GH}\;\mathrm{and}\;\mathrm{SED}\;\mathrm{and}\;\mathrm{trait}\;\mathrm{ratios}$$

with e and n being the number of environments and N levels, respectively, and V_G,_ V_G×E_, V_G×N_ and V_R_ being the variance components genotype, genotype*environment, genotype*N level and residual variance, calculated with SAS procedure VARCOMP according to the respective models:

Y=G+E+N+GxE+GxN+εfortraitsGPC,GH,andSED

and:

Y=G+E+εfortraitsGPC_ratio,GH_ratioandSED_ratio,

with Y, G, E, N, G×E, G×N, ε being the trait value, and the fixed effects genotype, environment, N level, genotype*environment, genotype*N level and residual variance, respectively. The analysis of variance was computed using the SAS procedure GLM applying the same general linear models as used for analyzing variance components as stated before.

Genetic relatedness among the 359 SW84 lines and with the three parents of the population was calculated as genetic similarity with SAS procedure DISTANCE, based on simple matching comparisons between the three possible genotype states across 4,096 informative SNPs. A graphical representation of genetic relatedness was carried out using a principal component analysis with SAS procedure PRINCOMP, based on the computed genetic similarity matrix.

### QTL analysis

We applied four mixed models in order to detect QTL for quality traits as indicated in Table 1. Model 1 was carried out across population SW84 and across both N levels. Model 2 was carried out within families T84 and D84, respectively, but across N levels. Model 3 was carried out across both families but within N levels N0 and N1, respectively, and model 4 within families and within N levels. Per SNP marker, thus, nine model applications were calculated for GPC, GH and SED and three for the respective N ratio traits. LSMEANS per line were calculated with SAS procedure MIXED across environments and N levels for models 1 and 2 and across environments but separately for each N level for models 3 and 4, respectively, using the following mixed model:

Y=L+E+LxE+ε,

where Y is the trait, L is the fixed effect of the SW line, E the random effect of the environment and L×E the random interaction effect.

**Table 1:** List of four mixed models (1–4) applied to carry out marker-trait association (MTA) analysis, depending on N levels and families considered.

N level Fam	Across families	Within T84	Within D84	Sum of MTAs
**Across N levels**	**1** (7)	**2** (6)	**2** (2)	(15)
**Within N0**	**3** (6)	**4** (2)	**4** (1)	(9)
**Within N1**	**3** (7)	**4** (3)	**4** (3)	(13)
**Sum of MTAs**	(20)	(11)	(6)	(37)

Values in brackets indicate number of significant (P_BON_<0.05) marker-trait associations (MTAs) detected.

For QTL detection, multiple linear regression Würschum et al. [60] was applied following a two-step scheme as indicated by Maurer et al. [50]. At first, SNP cofactors were selected to reduce genetic noise, using SAS procedure GLMSELECT. Subsequently, calculation of marker-trait associations was performed with SAS procedure MIXED according to the following multiple regression model:

$$Y=\mu +SNP+Fam+SNPxFam+{Cofactors}_{>1cM}+\varepsilon ,$$

where μ is the intercept, SNP the fixed effect for the SNP genotype, Fam the fixed family effect and ε the residual error. Only SNP cofactors with a distance more than 1cM apart from the SNP under study were included in the model. Each SNP was treated as a quantitative score of the presence of exotic alleles, where homozygous elite, heterozygous and homozygous exote genotypes were translated into SNP scores of 0, 1 and 2, respectively. To achieve a full genotype data set, which is needed for multiple regression analysis, missing genotypes were estimated applying the mean imputation (MNI) approach [61]. P values of the association study were adjusted as p_Bon,_ according to the step-down Bonferroni method [62], implemented in SAS procedure MULTTEST. SNP main effects, statistically significant with p_Bon_<0.05 were accepted as marker-trait association (MTA). Significant MTAs were merged into a single QTL if peak markers of models were linked by less than 5 cM [51]. The exotic effect of substituting two elite wheat alleles against two exotic SNP alleles was defined as the slope of the respective regression model multiplied by two. The variance explained per SNP (R^2^_adj_) was calculated according to Utz et al. [63].

## Results

### Analysis of genetic variation, based on iSELECT SNP data

In total, 81,587 scored SNP markers of the wheat iSELECT chip were tested for polymorphism and segregation distortion. Out of 67,718 SNPs, with failure rates lower than 100%, 11,161 SNPs were polymorphic in at least one SW84 family and 4,968 SNPs were polymorphic across both families, and passed the chi-square test of non-distorted segregation. Out of those, 4,096 informative SNPs were polymorphic in both families and, simultaneously, could be assigned to chromosome positions based on Wang et al. [54]. The latter set of 4,096 informative SNPs was used for further analyses (S1 Table). This set was distributed between the A, B and D genomes with 1,956 (47.75%), 1,473 (35.96%) 667 (16.28%) markers, respectively. Most of the informative SNPs mapped to homeologous group 6 chromosomes (21.95%), while only 8.33% mapped to group 7. The overall average distance between markers of 0.85 cM varied from 0.28 (on chromosome 6B) to 3.19 cM (on chromosome 4D).

**Fig 1:**
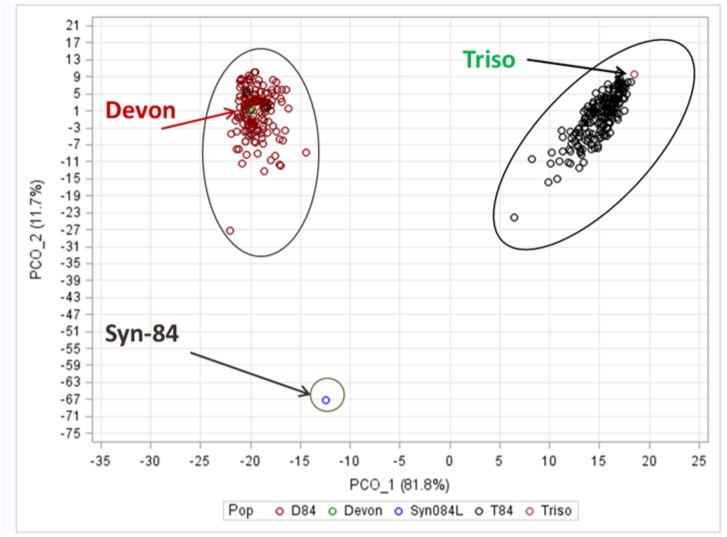
Principle component analysis based on calculation of genetic similarity between lines of SW84 families D84, T84 and the respective parents. D84 and T84 lines are depicted in red and black circles, respectively. The SW84 parents, Devon, Triso and Syn-84, are indicated by arrows. The explained variances of the first two principle components are given in percent.

The presence of exotic alleles across all three wheat genomes was calculated for each of the 359 SW84 lines. On average, SW lines covered 0.12 exotic alleles, varying from 0.014 to 0.296 (S1 Table). Genetic similarity (GS) was calculated between all 359 SW84 lines and the three founder parents (S2 Table). Family D84 showed a mean GS of 95.1% and 24.1% to Devon and Syn084L, respectively. Family T84 showed a mean GS of 94.3% and 25.3% to Triso and Syn084L, respectively. Genetic relatedness across population SW84 was assessed based on GS and is displayed in a principle component plot (Fig 1 and S2 Table). The first and second components of the principle component plot explained 81.8% and 11.7% of the total variation, respectively, and clearly separated the two families D84 and T84, placing the recurrent elite parents right into the respective family and the shared exotic donor between both families.

### Trait performance of population SW84

**Fig 2:**
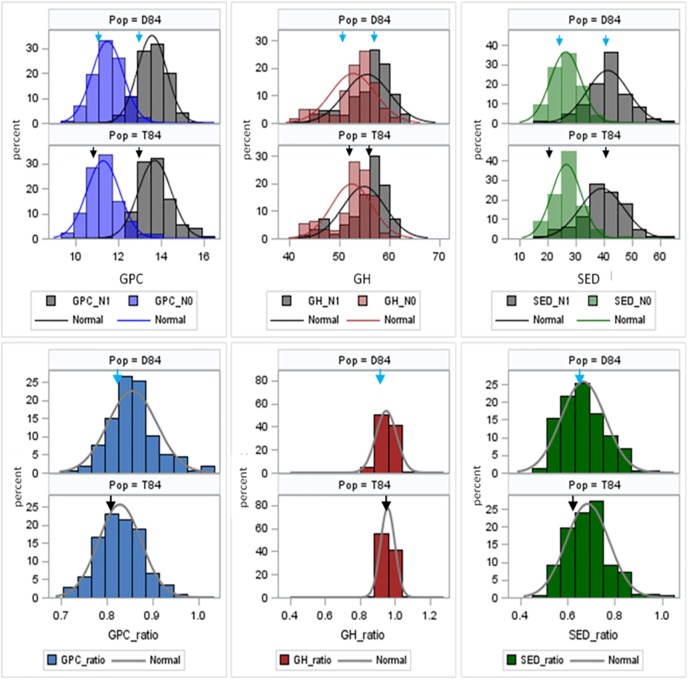
Frequency distribution of mean trait performance for GPC, GH, SED and the respective ratios, separated per family (D84 and T84) and color-coded for N0 and N1 treatments. The overlapping areas are colored in dark blue, dark red and dark green, respectively. Vertical arrows indicate mean values of controls Devon (blue) and Triso (black), respectively.

Phenotype raw data are stored in S1 Table. Descriptive statistics, calculated separately per family, environment and N level for six traits, are stored in S3 Table. Table 2 summarizes SW84 population performance within and across N levels From N0 to N1 levels, GPC, GH, and SED increased on average from 11.42 to 13.74%, 53.06 to 55.69%, and 26.66 to 40.30%, respectively. Thus, trait ratios between N levels amounted to mean values of 0.83, 0.96 and 0.62 for GPC_ratio, GH_ratio and SED_ratio, respectively. The highest coefficient of variation (CV) was found for SED_ratio with 33.13%, whereas the lowest CV was observed for GPC with 5.19%. The highest heritability (h^2^) was found for grain hardness across N levels (h^2^ = 93.48%). The three calculated trait ratios revealed heritabilities of 0%, indicating absence of genetic variance. Fig 2 displays frequency distributions for each trait separated by family and N level. Under low and high N supply, GPC and SED means of D84 and T84 were higher than the means of the recurrent parents Devon and Triso, respectively. Also, mean GH of D84 under low N supply was higher than the mean of the recurrent parent Devon. Also, mean GH of T84 under low and high N supply were higher than the means of the recurrent parent Triso. For all trait ratios, mean performances of families exceeded those of the recurrent parents. For all traits, investigated transgressive segregation could be identified where individual SW lines outperformed the recurrent elite parent.

**Table 2:** Trait performance of SW84 calculated across N levels and within N level, respectively.

			No of					CV	h^2^
Trait	Environments^a^	N level^b^	lines^c^	Mean^d^	Min^e^	Max^e^	SD^f^	(%)^g^	(%)^h^
GPC	D04 H04 H05	N0	359	11.42	9.51	13.85	0.75	6.56	47.54
(in%)	D04 H04 H05	N1	359	13.74	12.07	16.36	0.75	5.49	48.69
	D04 H04 H05	across	359	12.54	11.10	14.87	0.65	5.19	61.77
GPC_ratio	D04 H04 H05	across	359	0.83	0.53	1.25	0.09	11.28	0.03
GH	D04 H04	N0	359	53.06	39.00	64.00	4.43	8.35	80.34
(in %)	D04 H04	N1	359	55.69	40.50	66.00	4.66	8.36	84.90
	D04 H04	across	359	53.95	42.00	62.04	4.15	7.70	93.48
GH_ratio	D04 H04	across	359	0.96	0.45	1.51	0.07	7.17	0.00
SED	D04 H04	N0	327	26.66	16.69	45.50	5.36	20.09	43.16
(in ml)	D04 H04	N1	359	40.30	16.61	63.50	7.70	19.11	52.90
	D04 H04	across	359	36.23	24.50	56.33	4.96	13.68	53.17
SED_ratio	D04 H04	across	327	0.62	0.17	1.85	0.21	33.13	0.00

^a^ Environments: D04: Dikopshof 2004, H04: Hovedissen 2004, H05: Hovedissen 2005

^b^ N levels: N0: low nitrogen, N1: high nitrogen supply

^c^ Number of lines

^d^ Mean trait performance

^e^ Minimum and maximum trait performance

^f^ Standard deviation

^g^ Coefficient of variation

^h^ Heritability

### Trait correlations

Genetic correlations across N levels are shown in Table 3. The strongest negative correlation was observed between grain hardness and grain protein ratio (r = -0.53). The strongest positive correlation was found between grain protein content and sedimentation value (r = 0.87). Trait auto-correlations between N0 and N1 levels revealed high values. The highest auto-correlation between N0 and N1 was found for grain hardness (r = 0.84). In addition, grain protein content and sedimentation revealed moderate auto-correlations with r = 0.46 and r = 0.75, respectively.

**Table 3:** Genetic correlation (pearson’s correlation coefficient r) calculated across N levels between six traits studied.

	GPC	GH	SED	GPC_ratio	GH_ratio	SED_ratio
**GPC**	0.46***					
**GH**	0.57***	0.84***				
**SED**	0.87***	0.71***	0.75***			
**GPC_ratio**	*-0*.*13*	*-0*.53***	*-0*.38***	**n/a**		
**GH_ratio**	*-0*.*12*	*-0*.*17*	*-0*.*18*	0.32***	**n/a**	
**SED_ratio**	*-0*.*19*	*-0*.*15*	*-0*.21***	0.58***	0.67***	**n/a**

Values in bold, along the diagonal, indicate intra-trait correlations between N levels N0 and N1. Negative values are indicated in italics. Significance thresholds are

* P<0.05

** P<0.01

*** P<0.001. n/a: not available

### Mapping of QTL effects

Analysis of variance revealed significant effects (p<0.05) among genotypes, environments and N levels for grain protein content, grain hardness and sedimentation (S4 Table). In addition, a significant genotype effect was found for SED_ratio. Genotype interactions with environment or N level were not significant in any case. Significant environment effects were found for GH_ratio, GPC_ratio and SED_ratio.

**Fig 3:**
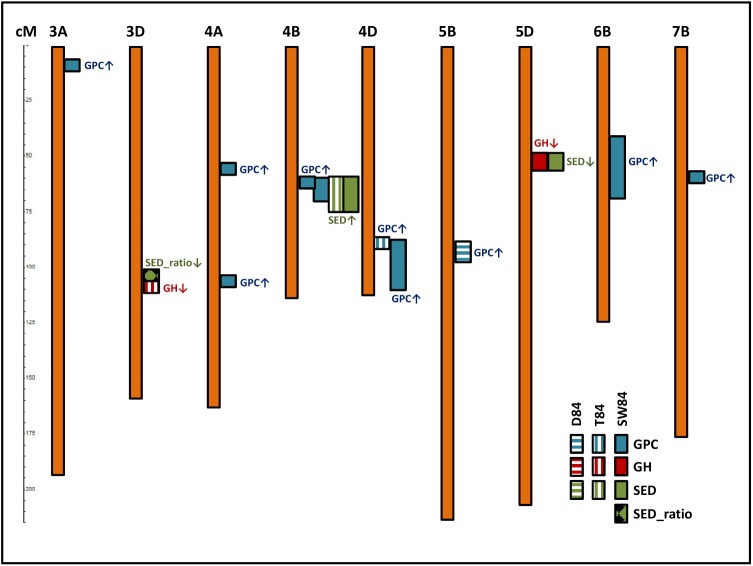
SNP map in cM locating 16 QTL for six traits detected across (SW84) and within families (D84 and T84). Trait abbreviations: GPC (Grain protein content), GH (grain hardness) and SED (sedimentation value), SED_ratio (sedimentation ratio, calculated between N0 and N1 levels). Arrowheads pointing up and down indicate increasing and decreasing trait effects of exotic QTL alleles, respectively.

Applying models 1–4 to SNP and phenotype data yielded 37 significant (P_BON_<0.05) independent marker-trait associations for grain protein content, grain hardness, sedimentation and sedimentation ratio (S5 Table and Figs A-D in S1 File). Most marker-trait associations were located based on model 3 (13x, i.e. across families and within N level), followed by model 4 (9x, within family and within N level), model 2 (8x, within family and across N levels) and model 1 (7x, across families and across N levels). Based on marker linkage and correspondence of marker effects, these associations were combined to QTL. In total, 16 QTL explaining genetic variation of four traits could be detected across or within the two families of SW84 and across or within the two N levels, respectively (Table 4 and Fig 3). Overall, 10, 2, 3 and 1 QTL, distributed among nine wheat chromosomes, were detected for GPC, GH, SED and SED_ratio, respectively. For GPC_ratio and GH_ratio no significant marker main effect could be detected. At 12 QTL (75%), favorable exotic alleles, improving trait performance, were identified. In the following, the QTL are presented for each trait separately.

**Table 4:** List of 16 grain quality QTL detected across or within two families of SW84 and across or within two N levels, respectively.

Trait^a^	QTL^b^	Model^c^	Fam^d^	N level^e^	Peak SNP^f^	Chr^g^	Pos (cM)^h^	QTL Range (cM)^i^	Exotic effect^j^	R_adj_^2^ ^k^	Candidate gene^l^
GPC	QGPC.SW84-3A	3	across	N1	CAP8_c1361_367	3A	12.04	12.04	0.66	6.5%	
	QGPC.SW84-4A.a	3	across	N1	IAAV4351	4A	58.38	58.38–61.91	0.51	5.1%	
	QGPC.SW84-4A.b	1	across	across	GENE_0439_61	4A	110.13	110.13	0.54	7.1%	
		3	across	N1	GENE_0439_61			108.72–110.13	0.56	6.2%	
	QGPC.SW84-4B.a	1	across	across	RAC875_c3299_226	4B	62.56	62.56	0.60	7.2%	
		2	T84	across	Tdurum_contig57516_269		61.84	61.84–62.56	0.74	10.9%	
		3	across	N0	RAC875_c3299_226		62.56	62.56–72.71	0.66	6.8%	
	QGPC.SW84-4B.b	3	across	N1	Ku_c33858_325	4B	72.52	62.56–72.52	0.63	5.2%	**GENE.1584_692** ^**1**^
	QGPC.T84-4D.a	4	T84	N1	D_contig75565_413	4D	87.18	87.18	1.09	8.8%	
	QGPC.SW84-4D.b	3	across	N0	BobWhite_c43880_73	4D	92.11	88.16–111.40	0.65	5.9%	**BobWhite_c47103_205** ^**1**^
	QGPC.D84-5B	4	D84	N1	Kukri_rep_c103366_421	5B	100.64	93.44–100.64	0.64	10.4%	
	QGPC.SW84-6B	1	across	across	Tdurum_contig61970_499	6B	56.98	39.11–71.97	0.70	10.4%	**NAM-B1** ^**2 + 3**^
		2	T84	across	Excalibur_c48499_250		57.40	57.40–57.48	0.75	8.5%	
		3	across	N0	Tdurum_contig61970_499		56.98	49.47–58.20	0.68	7.1%	
		3	across	N1	RAC875_c2730_1351		57.07	39.12–72.08	0.82	9.9%	
		4	T84	N1	RAC875_c2730_1351			49.47–71.97	1.02	9.5%	
		4	D84	N1	Tdurum_contig62040_1494		56.98	56.98	0.75	13.0%	
	QGPC.SW84-7B	3	across	N1	Kukri_c34345_122	7B	62.66	62.66	0.62	6.1%	** **
GH	QGH.T84-3D	2	T84	across	TA002219_1181	3D	113.56	113.51–113.56	*-3*.*58*	7.7%	* *
		4	T84	N0	JG_c34_270	3D	114.66	113.10–114.66	*-3*.*53*	7.1%	* *
	QGH.SW84-5D	1	across	across	D_GA8KES401AHAAQ_242	5D	51.93	51.93–56.32	*-8*.*98*	53.6%	***Ha Pinb*** ^3 + 4^
		2	T84	across	D_GA8KES401AHAAQ_242			51.93–56.32	*-8*.*65*	54.1%	
		2	D84	across	D_GA8KES401AHAAQ_242			51.93–54.46	*-9*.*26*	54.1%	
		3	across	N0	D_GA8KES401AHAAQ_242			51.93–56.32	*-8*.*81*	49.8%	
		3	across	N1	D_GA8KES401AHAAQ_242			51.93–56.32	*-9*.*02*	48.6%	
		4	T84	N0	D_GA8KES401AHAAQ_242			51.93–56.32	*-8*.*55*	52.0%	
		4	D84	N0	D_GA8KES401AHAAQ_242			51.93–54.46	*-9*.*18*	47.0%	
		4	T84	N1	D_GA8KES401AHAAQ_242			51.93–56.32	*-8*.*74*	49.5%	
		4	D84	N1	D_GA8KES401AHAAQ_242			51.93–54.46	*-4*.*67*	47.3%	
SED	QSED.SW84-4B.a	2	T84	across	Tdurum_contig57516_269	4B	61.84	61.84–75.65	7.31	9.8%	
		3	across	N0	TA006298_0500	4B	62.56	61.84–75.65	6.28	7.4%	
	QSED.SW84-4B.b	1	across	across	Ku_c33858_325	4B	72.52	61.84–75.65	7.18	7.6%	
	QSED.SW84-5D	1	across	across	D_GA8KES401AHAAQ_242	5D	51.93	51.93–54.46	*-11*.*26*	30.3%	***Ha Pinb***^**3 + 4**^**, Xbarc130** ^**5**^**, QSv.crc -5D** ^**6**^**, QSsd.caas-5D** ^**6+7**^
		2	T84	across	D_GA8KES401AHAAQ_242			51.93–54.46	*-10*.*50*	26.8%	
		2	D84	across	D_GA8KES401AHAAQ_242			51.93	*-12*.*21*	35.5%	
		3	across	N0	D_GA8KES401AHAAQ_242			51.93–54.46	*-9*.*94*	24.9%	
SED_ratio	QSEDr.SW84-3D	1	across	across	Kukri_c62907_121	3D	104.58	104.58	*-0*.*03*	0.4%	**Acc-2** ^**7+8**^

^a^ GPC: grain protein content; GH: grain hardness; SED sedimentation value

^b^ QTL name

^c^ model applied (see Table 1)

^d^ family studied

^e^ N level studied

^f^ peak SNP with highest -logP-value in QTL region

^g^ chromosomal location of peak SNP

^h^ position of peak SNP (in cM, following [54]

^i^ cM range of associated SNPs in a QTL region

^j^ exotic effect of substituting two elite alleles against two exotic alleles at peak SNP, defined as the difference between the homozygous exotic and the homozygous cultivated genotypes

^k^ explained adjusted variance (in%) of peak SNP effect

^l^ reference of candidate genes, potentially explaining the SNP effect: ^1^[27], ^2^[6], ^3^[19], ^4^[89], ^5^[90], ^6^[91], ^7^[32], ^8^[92].

#### Grain protein content (GPC)

QTL analysis revealed ten significant QTL for GPC (Table 4 and S5 Table). Three QTL were located with model 1 (across families and across N levels) whereas five and two QTL were exclusively located with models 3 (across families and within N levels) and 4 (within families and within N levels), respectively. The QTL are located on wheat chromosomes 3A, 4A, 4B, 4D, 5B, 6B and 7B. All exotic QTL alleles were associated with favorable effects, increasing grain protein content. The strongest favorable exotic allele effect was observed at QGPC.T84-4D where GPC was increased by 1.02% in family T84 under high N supply. The QTL QGPC.SW84-6B explained the maximum portion of genetic variance with 13.0% in family D84 under high N supply.

#### Grain hardness (GH)

For GH two significant QTL where located on chromosomes 3D and 5D. QGH.T84.3D was detected with models 2 (within family T84 and across N levels) and 4 (within family T84 and within N level N0). In contrast, QGH.SW84.5D was the only QTL detected with all four models and in all possible nine combinations (see Table 1). Both exotic QTL alleles were associated with unfavorable effects, reducing grain hardness. The strongest exotic allele effect was observed at QGH.SW84-5D where GH was reduced by -9.26% in family D84 across N levels. At this locus, the combinations D84 across N levels and T84 across N levels both explained the maximum portion of genetic variance with 54.1%.

#### Sedimentation value (SED)

For SED three significant QTL where located, two on chromosome 4B and one on chromosome 5D. Two QTL were located with model 1 (across families and across N levels) whereas one QTL, QSED.T84.3D, was only detected with models 2 (within family T84 and across N levels) and 3 (across families and within N level N0). Two exotic QTL alleles were associated with favorable effects, increasing sedimentation whereas one exotic QTL allele reduced sedimentation. The strongest favorable exotic allele effect was observed at QSED.SW84-4B.a where SED was increased by 7.31ml in family T84 across N levels. QSED.SW84-5D explained the maximum portion of genetic variance with 35.5% in family D84 across N levels.

#### Sedimentation ratio (SED_ratio)

For sedimentation ratio, the ratio of line performance between N0 and N1 levels, one QTL was detected on chromosome 3D. This QTL was detected with model 1 (across families and across N levels), decreased sedimentation ratio by 0.03%, and explained 0.4% of the genetic variance.

## Discussion

A number of AB-QTL studies have been published in wheat so far [64], [65], [66], [19], [46], [45], [67], [68], [47], [27]. Most studies were based on synthetic wild wheat donors, which were derived from crosses between durum wheat or wild emmer wheat and *Aegilops tauschii*, except [46], who used *T*. *macha* as the donor parent. Four AB-QTL studies investigated resistance to plant diseases, while three studies were used to analyze quality traits. All quality studies used winter wheat as the recurrent parent whereas the present study is the first using spring wheat cultivars as recipient and the first spring wheat AB-QTL study based on SNP genotyping. The number of 4,096 SNPs used in our study is similar to [47] who used 2,829 and 8,026 SNPs to map *Septoria tritici* blotch resistance in two winter wheat AB populations. Based on the improved resolution and ease of use we expect that future genome-wide association studies will preferentially make use of SNP markers. The markers used for the current analysis were not evenly distributed across the three wheat genomes, resulting in a strong bias against the D genome marker localization with a maximum gap of 53.24cM on chromosome 7D. This finding is reflecting a general tendency in genome studies of hexaploid wheat where the D genome is often underrepresented compared to the A and B genomes [69]. This holds true for [66] where chromosome 7D was also scarcely covered with SSR markers.

Near infrared reflectance spectroscopy, used in the present study to determine grain protein content, grain hardness and sedimentation value, has been used before for assessing quality parameters. It is a non-destructive method requiring no sample preparation. It is widely used as a method for texture assessment [70] and applied for economically analyzing product quality such as grains, fruits, meat, dairy products, beverages and others [71], [72]. De Sá et al. [73] referred to NIR as an analytical tool offering to analyze a large number of grains without intense efforts at a high level of repeatability. In QTL studies, NIR has been applied successfully to detect marker-trait associations in plants, for instance grain protein content and grain hardness [14], [28], [32], [74], [16], [17], [25], [34], nitrogen concentration [75], [24], [76], [77], [78], and sedimentation value [40]. These reports demonstrate the broad spectrum of applications NIR spectroscopy covers, in particular, for quality assessment in agriculture.

Breeding for grain quality comprises two major targets: to improve baking quality and to increase the nutritional value of wheat. Basic knowledge on the genetic relation of kernel characteristics, such as grain hardness and grain protein content, was also acquired in a wheat land race collection [79]. Negative correlations between grain yield and grain protein content, the latter being partly regulated through nitrogen fertilization and water availability is documented [80]. Considering these unfavorable relationships, improving end-use quality of wheat has proven to be demanding, both, in terms of knowledge of the causative genetic network and in terms of the requirements for various product types [81], [82]. Protein content is one of the major characteristics, which determine the commercial value of wheat and differentiate cultivars into quality classes. The functional *NAM-B1* allele was reported to show pleiotropy, resulting in increasing effects on protein and mineral content as well as on accelerating senescence [6]. The wild type allele of *NAM-B1* is assumed lost during domestication or during the nineteenth century at the latest. Nevertheless, the wild type allele was traced in some spring type bread wheat accessions of Fennoscandian origin [79].

The present study has located new exotic QTL alleles involved in and contributing to improving end-use quality (see Table 4). Several QTL are already reported in previous QTL studies, however other QTL are newly identified. In the following these QTL are discussed trait-wise and compared to other grain quality QTL studies in wheat.

### Grain protein content (GPC)

In the present study, GPC showed a high positive correlation across N levels with SED (r = 0.87). This finding corroborates with [19] where GPC also revealed a moderate positive correlation with SED in the winter wheat population B22. In the present study, heritability of GPC reached 0.62, calculated across N levels, being higher than reported in [32] but lower than reported in [83]. In total ten QTL controlling grain protein content were located in our study. In all cases, the exotic donor allele contributed to an increase of GPC, the condition selected in wheat breeding to improve baking quality. The QTL QGPC.SW84-6B, identified with marker Tdurum_contig61970_499, most likely corresponds to the gene *grain protein content B1* (*Gpc-B1)*, located on the short arm of chromosome 6B. This QTL was identified by [6] as the NAC transcription factor *NAM-B1*. In the present study, the exotic allele was associated with an increase in grain protein content as observed by [6] and [19]. This QTL was also mapped by [18], [74], [16], [25], [84] and [85], indicating that *Gpc-B1* presumably acts as a major factor to regulate high protein content in wheat.

The QTL found on the short arm of chromosome 3A may correspond to the QTL detected by [86] and [20]. In addition, [19] and [20] also found a QTL for GPC on the long arm of chromosome 3A. Further QTL controlling grain protein content were located on chromosomes 4A, 4B, 4D and 7B. These QTL may correspond to QTL mapped by [83], [17], [19], [20], [87], [85] and [27].

### Grain hardness (GH)

Grain hardness is a criterion for sorting wheat into market classes, such as hard and soft. In the present study, GH showed a high positive correlation across N levels with SED (r = 0.71). Calculated across N levels, heritability of GH reached 0.93, being higher than reported in [32] but similar to [83]. Two QTL controlling grain hardness were located in our study. In both cases, the exotic donor allele contributed to a decrease of GH, giving rise to softer grains, used to produce cookies, cakes and pastries but not yeast-leavened breads [88]. We identified a major cluster of marker-trait associations on chromosome arm 5DS, QGH.SW84-5D, which was omnipresent across families and N levels but also within each combination of family and N level. This QTL is in accordance with the studies of [83], [66] and [19]. The QTL most likely corresponds to the *Ha* locus where *puroindoline a (Pina)* and *puroindoline b (Pinb)* are located [19], [89]. In our study, the exotic QTL allele was associated with a reduction of both, GH and SED. This finding is further substantiated by the positive correlation observed between SED and GH. The co-localization of QTL for both traits may be attributed to pleiotropy effects of the *Pina* and *Pinb* genes. Also [84] identified a QTL for GH on the long arm of 5D.

### Sedimentation value (SED)

In the present study, SED showed a high positive correlation across N levels, both, with GPC (r = 0.87) and GH (r = 0.71). Calculated across N levels, heritability of SED reached 0.53 in the present study, being lower than reported in [40], [83] and [32]. In total four QTL, controlling sedimentation, were located in our study. In three cases, the exotic donor allele contributed to an increase of sedimentation whereas at QSED.SW84-5D the exotic QTL allele was associated with a decrease of GH. At the latter QTL a cluster of marker-trait associations was identified, again pointing to an omnipresent major QTL effect, which is present regardless of the family and the N level studied. This QTL was also mapped by [83], [32], [84], [39], [89], [90], [91] and [19], where the latter also identified a trait reducing, negative effect of the exotic donor allele. As stated before, this QTL most likely corresponds to the *Ha* locus where *puroindoline a (Pina)* and *puroindoline b (Pinb)* are located [19], [89]. The QTL may exert pleiotropic effects on both traits, GH and SED. It has to be tested in follow up studies with segregating near-isogeneic lines (NILs), if both effects can be separated through genetic recombination.

### Sedimentation ratio (SED_ratio)

Only one QTL was detected with a small effect on SED_ratio. The best blast hit for the respective marker was found for a *cytosolic acetyl-CoA carboxylase (Acc-2)* located on the long arm of group 3 chromosomes [32], [92]. No QTL were detected for any further ratio trait calculated. This finding may be the result of increasing the residual variance by adding error variances from two independent measurements through calculating ratios between line performances under low and high N levels. A similar finding was reported by [93] when carrying out a QTL analysis with water stress indices derived from testing barley introgression lines under two water regimes. In a study on mathematically-derived traits in QTL mapping [94] pointed out that an increased complexity of the genetic architecture of derived traits (e.g. stress indices) may reduce the power of QTL detection. We, thus, recommend running GWAS studies for each treatment separately, in addition to a joint GWAS analysis across treatments, to identify treatment-specific marker-trait associations. We have implemented these additional GWAS studies in our models 2 to 4.

Previous QTL studies using wild progenitors also detected new exotic QTL alleles with no corresponding gene published beforehand. For instance, von Korff et al. [95], studying wild-barley advanced backcross lines reported on three new QTL controlling brittleness and on ten new QTL controlling ears per m^2^. Narasimhamoorthy et al. [66] detected an unknown hard winter wheat QTL reported for kernel hardness, where the favorable allele was contributed by the exotic parent. Also, Naz et al. [47] detected eight and five so far unreported exotic QTL alleles involved in resistance against *Septoria tritici* blotch when studying two exotic wheat advanced backcross populations. Here, 53% of the exotic alleles proved to be beneficial against *Septoria tritici* blotch. This finding corresponds to Kunert et al. [19] who found in one of these populations that 36.8% of the QTL detected for milling and baking quality revealed favorable exotic wheat alleles. Exotic wheat and wild barley resources, thus, proved to carry novel allelic variants. To some extent, they prove to be beneficial and ready to be transferred to the elite gene pool to increase trait performance in cultivated crops.

### Comparison of detected marker-trait associations between models 1–4

We studied baking quality traits in a tri-parental advanced backcross spring wheat population, SW84, under two nitrogen fertilization levels. In order to distinguish common effects across families and N levels from effects within family and N level, we consecutively carried out genome-wide association studies with four separate models 1–4. For this, we only used SNP markers, which segregated in both families simultaneously. As indicated in Table 1, most effects were detected with model 3 (13 effects across families & within N level), followed by model 4 (nine effects within family & within N level), model 2 (eight effects within family & across N levels) and model 1 (seven effects across families & across N levels). Remarkably, the major grain hardness QTL QGH.SW84-5D, explaining up to 54.1% of the variation was the only QTL detected with all four models and all nine combinations simultaneously (see Table 4). As mentioned before, applying four models resulted in higher QTL detection rates than using calculated trait ratios. In addition, the QTL detection rates varied only slightly between the four models, giving rise to 20, 15 and 7 marker-trait associations, which can be classified as stable across families, across N levels and across both factors simultaneously. This information may be very important for breeding purposes as well as for characterization of gene effects, since valuable information about the stability of the QTL effect is connected to the data. From a breeding perspective, those QTL, which are stable across families and N levels may be preferred to support a broader utilization. The major QTL QGPC.SW84-6B and QSED.SW84-4B.b may be good examples where the exotic alleles are associated with an increase of 0.70% and 7.18 ml in grain protein content and sedimentation value, respectively.

Comparing individual family effects, more effects in family T84 than in family D84 were detected (11 versus 6, see Table 1). Since both families of SW84 share the same exotic donor parent, the family preference may be attributed to the larger population size in T84 than in D84 (205 versus 154 SW lines). In addition, the recipient Triso may possess a stronger genetic contrast to the exotic donor Syn084L than Devon regarding the control of the studied quality traits. In future, we, thus, recommend using larger populations and more donor accessions to increase the chance of finding new and favorable exotic QTL to be used in wheat breeding. An ideal population for this purpose may be the nested association mapping populations as demonstrated in corn and barley [96], [50].

Comparing individual N level effects, more effects under the high N level N1 than under the low N level N0 were detected (13 versus 9, see Table 1). This phenomenon may be attributed to the increased residual variance under stress, i.e. N level N0, as indicated by higher coefficients of variation and lower heritabilities under N0 compared to N1 for all three traits studied (see Table 2). We, thus, recommend to preferentially relying on data from unstressed field experiments in breeding experiments, if the stress performance per se is not a target for selection. This way, a higher level of reproducibility and a higher number of QTL can be expected.

## Conclusions

Our genome-wide association study using the exotic tri-parental advanced backcross wheat population SW84 has revealed a number of exotic QTL alleles, which control the genetic regulation of the wheat major quality traits grain protein content, grain hardness and sedimentation value. A remarkably high number of favorable exotic QTL alleles, one corresponding to the candidate gene *Nam-B1*, were discovered for grain protein content and sedimentation value. In future, the exotic *NAM-B1* allele on chromosome 6B and the remaining, so far, unknown favorable exotic QTL alleles will be validated in independent field experiments. Subsequently, validated favorable alleles will be used in wheat breeding programs to improve grain quality. For this, near-isogenic lines will be developed based on existing SW84 lines, which cover the exotic target QTL alleles. In addition, the SW84 lines may be directly used to introgress the exotic target QTL region into the genomic background of new elite spring wheat and winter wheat cultivars. The available set of iSELECT SNP data may be an ideal starting point to select improved offspring lines based on marker-assisted selection. Our study serves as a model how favorable exotic genes can be localized and, subsequently, utilized in plant breeding to selectively expand the genetic diversity of the elite wheat gene pool. We expect that, in future, utilizing exotic germplasm in wheat breeding programs will be extended and substantially accelerated by adopting the up-coming complete reference genome sequence of wheat [97].

## Supporting information

S1 Table Genotype and phenotype raw data per SW84 line. (XLSX)

S2 Table Genetic similarity (GS) between SW84 lines and principle components (PCOs). (XLSX)

S3 Table Descriptive statistics calculated per family, environment and N level. (XLSX)

S4 Table Analysis of variance (ANOVA) for six traits studied. (XLSX)

S5 Table GWAS output for marker-trait associations per SNP. (XLSX)

S1 File Fig A: GWAS Manhattan plot for grain protein content (GPC). Fig B: GWAS Manhattan plot for grain hardness (GH). Fig C: GWAS Manhattan plot for grain sedimentation (SED). Fig D: GWAS Manhattan plot for sedimentation ratio (SED_ratio).
(DOCX)
