# Supplementary material for: Exotic QTL improve grain quality in the tri-parental wheat population SW84
Source: PLoS One. 2017 Jul 7;12(7):e0179851. doi: 10.1371/journal.pone.0179851 (PMC5501409; doi:10.1371/journal.pone.0179851)
Supplement: S1 File — Fig A: GWAS Manhattan plot for grain protein content (GPC). Fig B: GWAS Manhattan plot for grain hardness (GH). Fig C: GWAS Manhattan plot for grain sedimentation (SED). Fig D: GWAS Manhattan plot for sedimentation ratio (SED_ratio). (DOCX) [file pone.0179851.s006.docx]

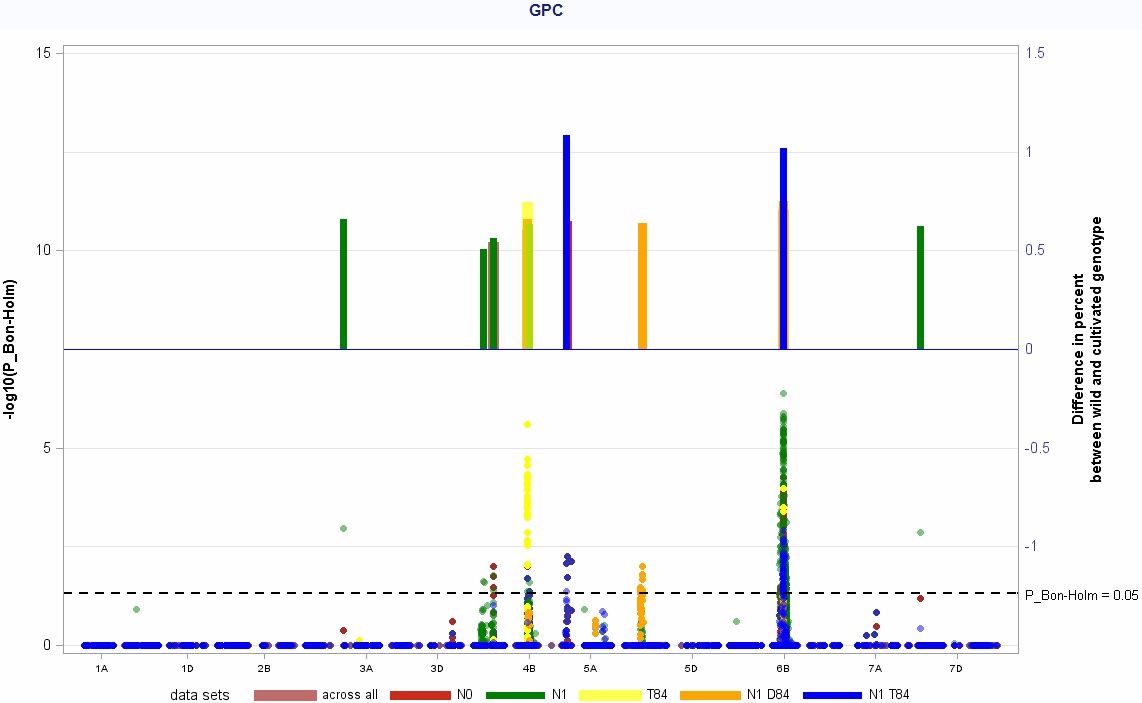


**Fig. A in S1 file: GWAS Manhattan plot for grain protein content (GPC).**

Lower part of graph and left y-axis: P_BON_ values of SNP marker-trait associations plotted against the chromosomal SNP position taken from Wang et al. (2014). Dots above the grey dashed threshold line represent significant (P_BON_<0.05) marker-trait associations.

Upper part of graph and right y-axis: SNP effect (in %) at significant marker-trait associations, where the two elite alleles are substituted against the two exotic alleles (see Table 4 for details).

Color coding of dots and lines indicate GWAS model used, i.e. across or within families and N levels, respectively.


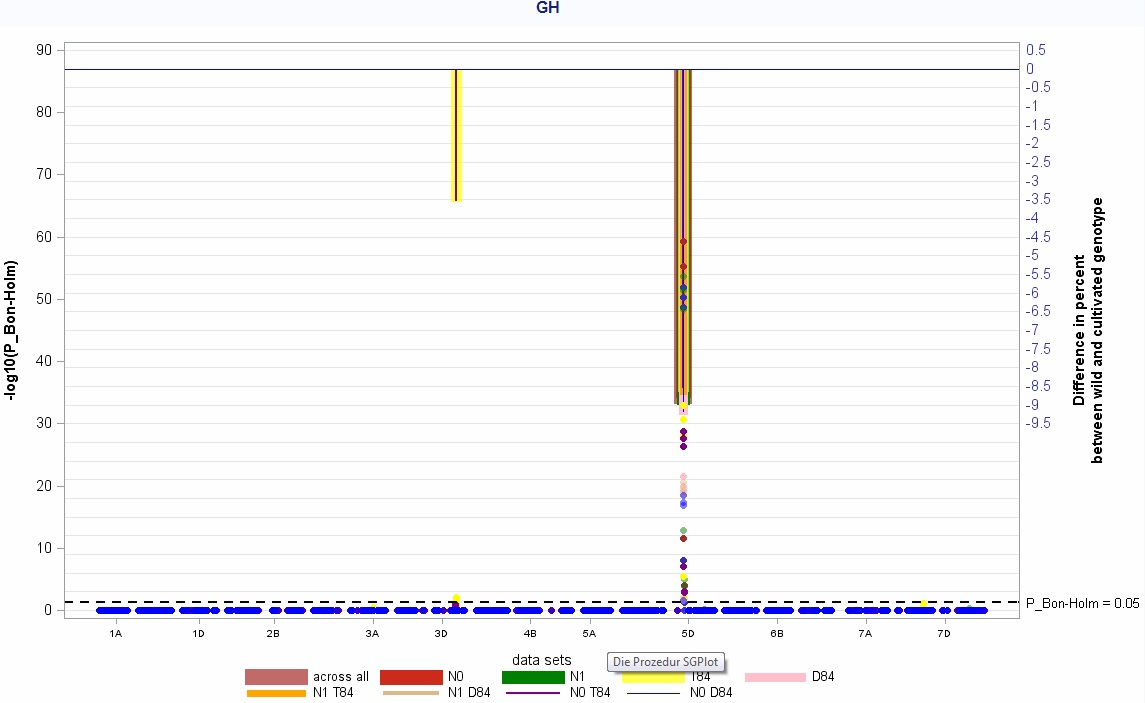


**Fig. B in S1 file: GWAS Manhattan plot for grain hardness (GH).**

Lower part of graph and left y-axis: P_BON_ values of SNP marker-trait associations plotted against the chromosomal SNP position taken from Wang et al. (2014). Dots above the grey dashed threshold line represent significant (P_BON_<0.05) marker-trait associations.

Upper part of graph and right y-axis: SNP effect (in %) at significant marker-trait associations, where the two elite alleles are substituted against the two exotic alleles (see Table 4 for details).

Color coding of dots and lines indicate GWAS model used, i.e. across or within families and N levels, respectively.


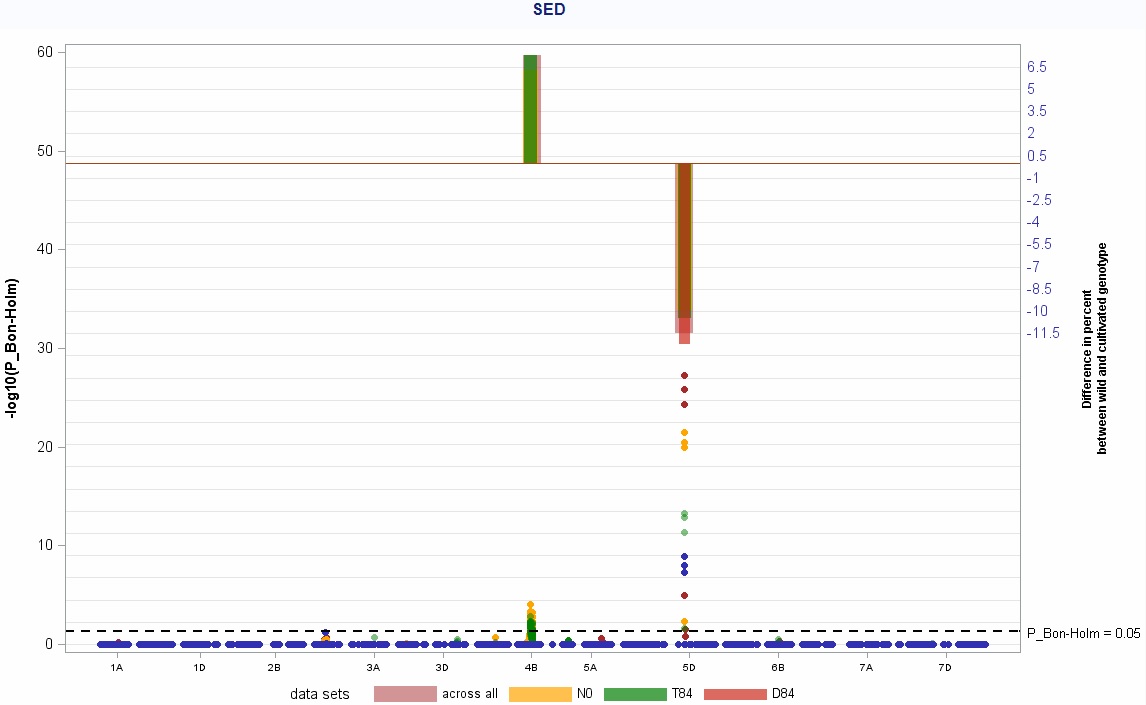


**Fig. C in S1 file: GWAS Manhattan plot for grain sedimentation (SED).**

Lower part of graph and left y-axis: P_BON_ values of SNP marker-trait associations plotted against the chromosomal SNP position taken from Wang et al. (2014). Dots above the grey dashed threshold line represent significant (P_BON_<0.05) marker-trait associations.

Upper part of graph and right y-axis: SNP effect (in ml) at significant marker-trait associations, where the two elite alleles are substituted against the two exotic alleles (see Table 4 for details).

Color coding of dots and lines indicate GWAS model used, i.e. across or within families and N levels, respectively.


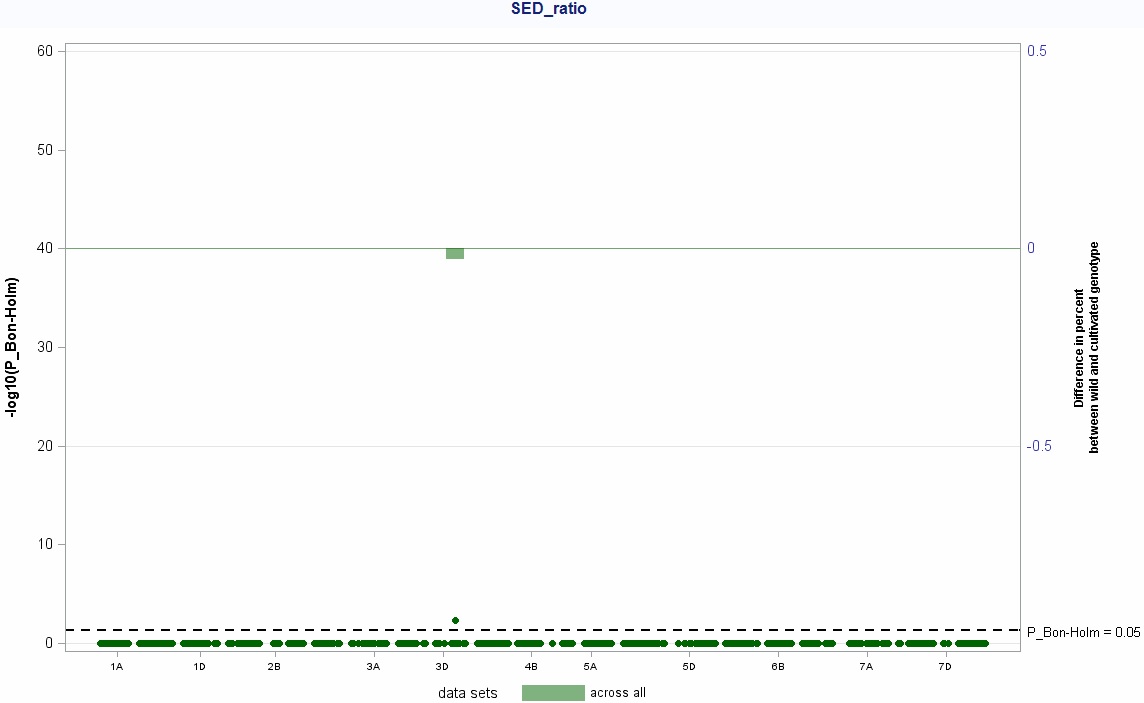


**Fig. D in S1 file: GWAS Manhattan plot for sedimentation ration (SED_ratio).**

Lower part of graph and left y-axis: P_BON_ values of SNP marker-trait associations plotted against the chromosomal SNP position taken from Wang et al. (2014). Dots above the grey dashed threshold line represent significant (P_BON_<0.05) marker-trait associations.

Upper part of graph and right y-axis: SNP effect at significant marker-trait associations, where the two elite alleles are substituted against the two exotic alleles (see Table 4 for details).

Color coding of dots and lines indicate GWAS model used, i.e. across or within families and N levels, respectively.
